# Associations between avocado intake and measures of glucose and insulin homeostasis in Hispanic individuals with and without type 2 diabetes: Results from the Hispanic Community Health Study/Study of Latinos (HCHS/SOL)

**DOI:** 10.1016/j.numecd.2023.08.002

**Published:** 2023-08-18

**Authors:** MacKenzie K. Senn, Mark O. Goodarzi, Gautam Ramesh, Matthew A. Allison, Mariaelisa Graff, Kristin L. Young, Gregory A. Talavera, Amanda C. McClain, Tanya P. Garcia, Jerome I. Rotter, Alexis C. Wood

**Affiliations:** a USDA/ARS Children’s Nutrition Research Center, Baylor College of Medicine, 1100 Bates Avenue Houston, TX 77030, USA; b The University of Texas Health Science Center at Houston School of Public Health, 1200 Pressler Street, Houston, TX 77030, USA; c Division of Endocrinology, Diabetes, and Metabolism, Department of Medicine, Cedars-Sinai Medical Center, Los Angeles, CA 90048, USA; d School of Medicine, University of California, La Jolla, San Diego, CA 92037, USA; e Division of Preventive Medicine, Department of Family Medicine, University of California, La Jolla, San Diego, CA 92037, USA; f Department of Epidemiology, Gillings School of Global Public Health, University of North Carolina, Chapel Hill, NC 27599, USA; g Department of Psychology, San Diego State University, San Diego, CA 92182, USA; h School of Exercise and Nutritional Sciences, San Diego State University, San Diego, CA 92182, USA; i Department of Biostatistics, Gillings School of Global Public Health, University of North Carolina, Chapel Hill, NC 27599, USA; j The Institute for Translational Genomics and Population Sciences, Department of Pediatrics, The Lundquist Institute for Biomedical Innovation at Harbor-UCLA Medical Center, Torrance, CA, USA

**Keywords:** Avocado, Diet, HbA1c, Type 2 diabetes, Postprandial, Insulin homeostasis

## Abstract

**Background and Aims:**

To investigate associations between avocado intake and glycemia in adults with Hispanic/Latino ancestry.

**Methods and Results:**

The associations of avocado intake with measures of insulin and glucose homeostasis were evaluated in a cross-sectional analysis of up to 14,591 Hispanic/Latino adults, using measures of: average glucose levels (hemoglobin A1c; HbA1c), fasting glucose and insulin, glucose and insulin levels after an oral glucose tolerance test (OGTT), and calculated measures of insulin resistance (HOMA-IR, and HOMA-*%*β), and insulinogenic index. Associations were assessed using multivariable linear regression models, which controlled for sociodemographic factors and health behaviors, and which were stratified by dysglycemia status.

In those with normoglycemia, avocado intake was associated with a higher insulinogenic index (β = 0.17 ± 0.07, *P* = 0.02). In those with T2D (treated and untreated), avocado intake was associated with lower hemoglobin A1c (HbA1c; β = −0.36 ± 0.21, *P* = 0.02), and lower fasting glucose (β = −0.27 ± 0.12, *P* = 0.02). In the those with untreated T2D, avocado intake was additionally associated with HOMA-%β (β = 0.39 ± 0.19, *P* = 0.04), higher insulin values 2-h after an oral glucose load (β = 0.62 ± 0.23, *P* = 0.01), and a higher insulinogenic index (β = 0.42 ± 0.18, *P* = 0.02). No associations were observed in participants with prediabetes.

**Conclusions:**

We observed an association of avocado intake with better glucose/insulin homeostasis, especially in those with T2D.

## 1. Introduction

The prevalence of T2D is 66% higher in US Hispanic/Latino persons than in their Non-Hispanic White (NHW) counterparts [1]. The age and sex-standardized prevalence for impaired fasting glucose (excluding those with a diagnosis of T2D) is 31.6% in Mexican-Americans compared to 26.1% in NHW. In addition, the proportions of individuals with impaired glucose tolerance and insulin resistance show similar disparities by race/ethnicity [2]. Therefore, identifying factors associated with dysglycemia and T2D risk in individuals with Hispanic/Latino ancestry remains an important strategy for reducing health disparities in the US.

Diet plays a strong role in both the prevention and treatment of T2D [3]. Although the American Diabetes Association (ADA) tailors dietary advice to whether an individual has T2D or not, for both groups the mainstay of recommendations include reducing overall fat and energy intake, increasing fiber intake, focusing on whole grains, and considering meals/snacks with a low glycemic load [4]. Current ADA advice represents a departure from earlier recommendations, in that the recommendation to include a set amount of monounsaturated fats (MUFA) as a proportion of overall energy intake, included in 2002 and 2004, has been removed [5,6]. The American Association of Clinical Endocrinologists also exclude MUFA from dietary recommendations to prevent T2D [7]. This is at odds with the Diabetes and Nutrition Study Group of the European Association for the Study of Diabetes [8], and The British Diabetic Association (Diabetes UK [9]), who recommend that MUFA may provide 10–20% total energy (provided that fat intake does not exceed 35% total energy), and to “include healthy fats in your diet”, respectively [8,9]. Overall, there is a lack of consensus on the role of MUFA in T2D risk.

The conflicting guidelines regarding the role of MUFA in T2D prevention may, in part, reflect the complexity of the relationship of MUFA intake to T2D risk. At a population level, data are conflicting as to the relationship between MUFA intake and dysglycemia [10–12]. This may be because the relationship is dependent upon the extent of impaired glucose tolerance; in a recent meta-analysis MUFA intake was associated with lower hemoglobin A1c (HbA1c) in individuals with abnormal glucose metabolism [13], but a larger association was seen in those with T2D, as well as significant inverse associations with fasting glucose and insulin resistance [14].

Avocados are a good source of MUFA, with a typical serving size (one-half of an avocado) providing 6.7 g of MUFA. Data from small intervention studies have suggested that consuming avocado can improve glycemia. In one randomized controlled trial (RCT) of 31 non-smoking adults with overweight or obesity, participants consuming either one-half, or one whole, of an avocado at breakfast had significantly lower glycemic and insulinemic responses to their meal, than participants consuming an isocaloric avocado-free control meal [15]. After meal consumption, peak postprandial glucose concentrations were ~1 mmol/L lower in those who consumed either amount of avocado, with an effect of similar magnitude seen on insulin responses [15]. In a similar randomized cross-over trial of 26 adults with overweight, when the salad and cookies of a mixed meal were exchanged for an isocaloric portion of avocado, insulin levels 30 min following the meal ingestion were ~20 μIU/ml lower [16]. However, no effect on hemoglobin A1c was found in either trial [15,16], which is likely due to HbA1c representing average glucose levels over the preceding three-months, which cannot be changed retrospectively. Only one longer-term observational study has, to our knowledge, examined whether avocado intake is associated with dysglycemia, reporting a lower incidence of T2D among avocado consumers compared to non-consumers [17]. However, lending weight to the notion that diet-glycemia associations differ by glycemia status ([13,14]), this finding was only significant in those with prediabetes at baseline, not in those with normoglycemia [17].

Therefore, whether avocado intake is associated with glycemia, and if so in whom, remains unclear. Yet, if avocado does convey beneficial effects on glycemia, Hispanic/Latino persons could stand to gain the most benefit. Hispanic/Latino individuals have the highest avocado intake in the U.S. [18,19], with consumers typically eating more than one-half of a medium-sized avocado daily on average [20].

Thus, the goal of the current analyses was to assess the association between avocado intake on average glucose levels (HbA1c), and measures of glucose and insulin homeostasis, including: insulin sensitivity/resistance (HOMAIR) and β-cell functioning (HOMA-%β), insulin and glucose values both at fasting, and 2-h after an oral glucose challenge (postprandial measures) administered as part of an oral glucose tolerance test (OGTT), and as the interplay between insulin and glucose changes in response to the glucose challenge (insulinogenic index). We hypothesized that avocado intake would be associated with lower HbA1c, and lower postprandial insulin and glucose levels - findings we expected to be stronger in those with dysglycemia. To test our hypotheses, we used cross-sectional data on a large, nationally-representative sample of 14,591 US adults with Hispanic/Latino ancestry (N = 11,996 for postprandial traits) participating in the Hispanic Community Health Study/Study of Latinos (HCHS/SOL).

## 2. Methods

### 2.1. Participants

HCHS/SOL is a community-based prospective cohort study of Hispanics/Latinos aimed at describing the prevalence of risk and protective factors for chronic conditions (e.g., cardiovascular disease (CVD), diabetes and pulmonary disease), and to quantify all-cause mortality, fatal and non-fatal CVD and pulmonary disease, and pulmonary disease exacerbation over time [21,22]. HCHS/SOL recruited 16,415 self-identified Hispanic/Latino persons between 2008 and 2011, aged 18–74 years at baseline, across four U.S. field centers (Chicago, IL; Miami, FL; Bronx, NY; San Diego, CA).

The HCHS/SOL study was approved by the Institutional Review Boards at all HCHS/SOL participating institutions and all participants provided informed written consent. The current analyses were reviewed by the Institutional Review Board at Baylor College of Medicine and approved under exempt status.

### 2.2. Sampling design and study sample

**Figure 1:**
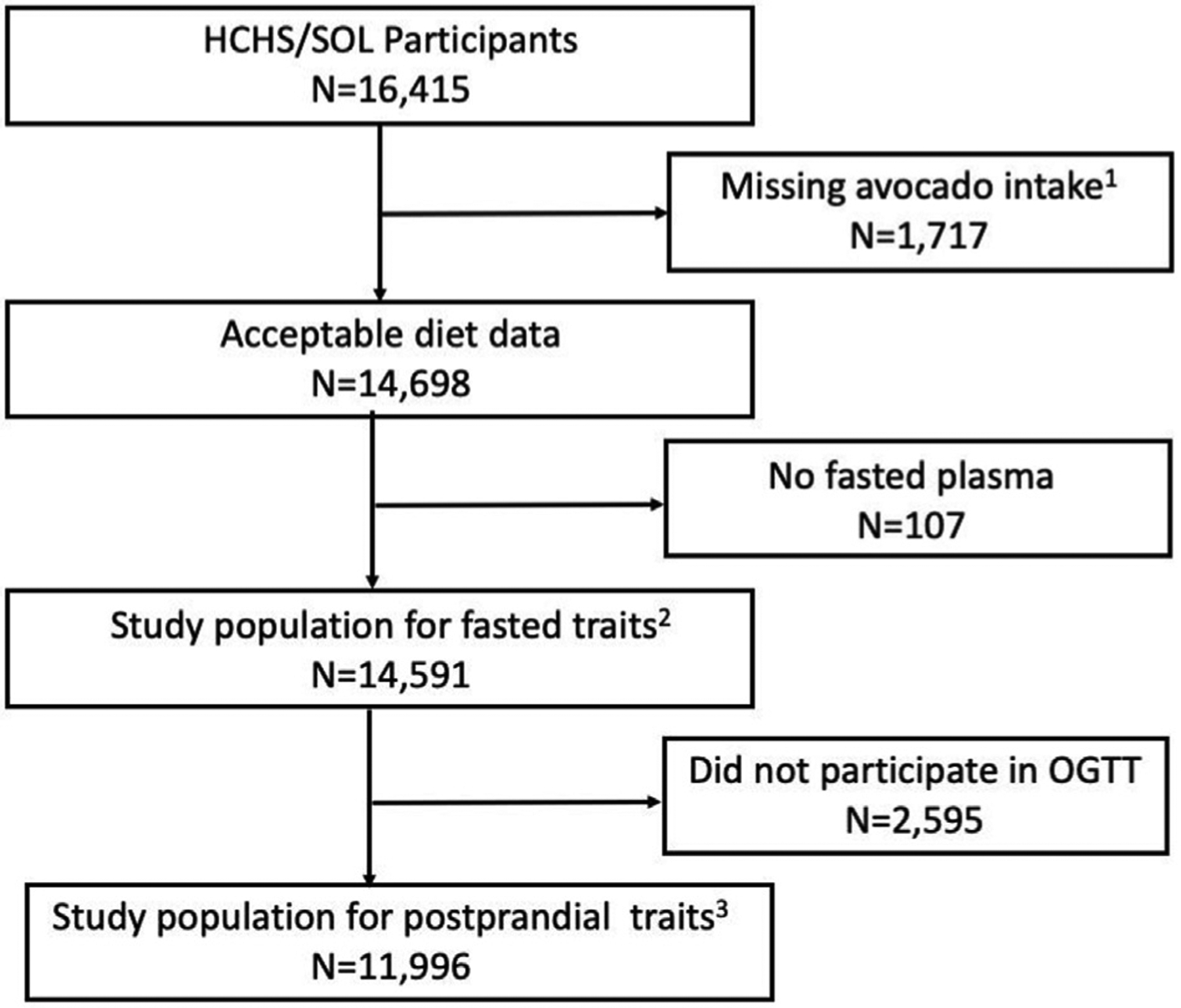
Participant Flow Diagram Abbreviations: HCHS/SOL: Hispanic Community Health Study/Study of Latinos; OGTT: Oral glucose tolerance test; N: Frequency. ^1^ Missing avocado intake data on either or both days ^2^ Fasted traits include insulin, glucose, and hemoglobin A1c (HbA1c) ^3^ Postprandial traits include insulin and glucose 2-h after an oral glucose load, and the differences between fasting and postprandial values for each of glucose and insulin.

The study design for HCHS/SOL is described in detail elsewhere [21]. A stratified two-stage area probability sample of household addresses was selected in each of the four field centers. The first sampling stage randomly selected census block groups with stratification based on Hispanic/Latino concentration and proportion of high/low socio-economic status. The second sampling stage randomly selected households, with stratification, from US Postal Service registries that covered the randomly selected census block groups. Both stages oversampled certain strata to increase the likelihood that a selected address yielded a Hispanic/Latino household. After households were sampled, in-person or telephone contacts were made to screen eligible households and to roster its members. Lastly, although the full age range (18–74 years) was represented at baseline, the study oversampled the 45–74 y age group (n = 9,714, 59.2%) to facilitate examination of target outcomes. As a result, participants included in the HCHS/SOL cohort were selected with unequal probabilities of selection, and HCHS/SOL sampling weights accounted for non-response relative to the sampling frame, trimmed to handle extreme values being overly influential in the analyses, and were calibrated to the 2010 U S. Census according to age, sex and Hispanic heritage [21]. Participants were excluded from the current analyses if they were missing data on avocado intake, or on all measures of glycemia and insulin homeostasis (N = 1,824; Fig. 1). This resulted in a final sample of up to N = 14,591 for analysis (Fig. 1).

### 2.3. Measures

The baseline in-person clinic visit included a comprehensive suite of assessments. These included biological (e.g., anthropometrics), behavioral (e.g., dietary intake and self-reported physical activity [23]), and socio-demographic measures [21,22]. Participants without a previous diagnosis of diabetes, and with FPG <150 mg/dL (as assessed on site via a SureStepPro Glucose Meter), also completed a standard 75 g 2-h oral glucose tolerance test (2 h OGTT) during the baseline clinic exam (N = 11,996; Fig. 1) [24].

#### 2.3.1. Glucose and insulin homeostasis

For fasting traits, blood samples were collected after an 8-h fast [24]. For postprandial traits, blood was collected 120 min after the ingestion of a 75 g glucose load [25].

HbA1c (fasting only) was measured from whole blood by assay using a Tosoh G7 Automated High-Performance Liquid Chromatography (HPLC) Analyzer (Tosoh Bioscience, Inc., San Francisco, CA) [25,26]. HbA1c is presented both as the percentage of glycosylated hemoglobin (%HbA1c), and in mmol/mmol (SI units) calculated as glycosylated hemoglobin (mmol/mmol) = % HbA1c × 10.93) – 23.50 [27].

Glucose was measured in plasma on a Roche Modular P Chemistry Analyzer (Roche Diagnostics Corporation) using a hexokinase enzymatic method in mg/dL.

Insulin was measured with one of two commercial immunoassays; until October 2009, this was conducted by ELISA (Mercodia AB, Uppsala, Sweden), and after this time by sandwich immunoassay (Roche Elecsys 2010 Analyzer, Roche Diagnostics, Indianapolis, IN) [28,29]. To harmonize data, measures from the ELISA assay were calibrated by multiplying the value in mU/L ((1 mU/L = 6 pmol/L) by 1.00494 and deducting 1.4504 [i.e, (1.00494 × ELISA value in (mU/L)) – 1.4504].

Based on these insulin and glucose values, HOMA-IR was calculated as HOMA-IR = fasting glucose (mg/dL) * fasting insulin (mU/L)/405. HOMA-%β was calculated as HOMA-%β = 360 × fasting insulin (mU/L)/(fasting glucose (mg/dL – 63) [30]. The insulinogenic index was calculated as the change in insulin between fasting and 2-h after start of the OGTT, divided by the change in glucose over the same time frame [i.e., (2-h OGTT insulin (mU/L)– fasting insulin (mU/L))/(2-h OGTT glucose (mg/dL)– fasting insulin (mg/dL)).

#### 2.3.2. Diabetes status

T2D status was determined based on ADA criteria [31], i.e., fasting glucose ≥126 mg/dL, or glucose ≥200 mg/dL 2-h after an oral glucose load (2-h OGTT), or HbA1c ≥6.5%, or the participant self-reported a previous diagnosis of T2D or medication usage for T2D. Prediabetes was defined as fasting glucose range 100–125 mg/dL, or 2 h-OGTT glucose in range 140–199 mg/dL, or 5.7%≤ HbA1c<6.5%. Normoglycemia was defined as fasting glucose range < 100 mg/dL, post-OGTT glucose in range <140 mg/dL, and <5.7% HbA1c.

#### 2.3.3. Dietary intake

Participants completed two 24-h recalls, using a multipass technique, at an interval of 5–45 days apart. The first diet recall covered the 24-h period prior to initiating a fast for the baseline clinic visit during which time the blood draw was collected. Although 24-h recalls are subject to similar biases and error as all self-report nutrition data, validity coefficients suggest these are not higher in Hispanic/Latino data, including those from HCHS/SOL, than expected in other US populations [32,33]. Nutrient and food group values for reported intakes were derived from the Nutrition Data System for Research (NDSR [34]).

Avocado intake in servings/day and total energy intake (kcal/day) were derived from an average of the two 24-h recalls. A single serving of avocado in the NDSR database equates to one-third of a medium avocado, or 50 g [34]. Alcohol intake was estimated in grams/day as per NDSR [34]. A dietary quality score (used as a covariate) was assessed via scores on the alternative Healthy Eating Index-2010 (aHEI-2010), which sums decile scores for typical consumption across 11 dietary components: (1) vegetables without potatoes, (2) whole fruits, (3) whole grains, (4) long-chain (n-3) fats (EPA + DHA), (5) polyunsaturated fatty acids (PUFA), and (6) nuts and legumes (which are all positively scored), and (7) sugar sweetened beverages and fruit juices, (8) red/processed meat, (9) trans fats, (10) sodium, and (11) alcohol (which are all negatively scored). AHEI-2010 scores range from 0 to 110 with higher scores representing a healthier diet.

#### 2.3.4. Demographics

Age, sex, education level, smoking status, Hispanic/Latino heritage, nativity and preferred language were obtained through in-person interview with trained assessors.

#### 2.3.5. Acculturation

As done in previous epidemiological studies that include Hispanics/Latinos living in the US [35], an acculturation score was constructed from three proxy measures: nativity, years living in the US and preferred language of interview. U.S. Nativity and years in the U.S. were combined and scored as U.S.-born (3 points), foreign-born and lived in the U.S. at least 20 years (2 points), foreign-born and lived in the U.S. 10–19 years (1 point), or foreign-born and lived in the U.S. less than 10 years (0 points). A separate score was given for language spoken at home: English (2 points), English and Spanish (1 point), or Spanish language (0 points). The two scores were summed to obtain an acculturation score from 0 (least acculturated) to 5 (most acculturated) following previous analyses [36].

#### 2.3.6. Physical activity

Self-reported physical activity was assessed using an adapted version of the World Health Organization (WHO) Global Physical Activity Questionnaire [37], which is designed to ask about time spent in physical activity, and the type of activity conducted during an average week, across three life domains (work-related, transportation, and leisure/recreational). Time spent in moderate and vigorous physical activity was converted into metabolic equivalent of task units (METs).

#### 2.3.7. Anthropometric measures

Height and weight were measured by trained study staff. BMI was calculated as weight in kilograms (kg) divided by height in meters (m) squared (kg/m^2^).

### 2.4. Statistical analyses

All analyses were conducted using R software (version 4.0.5) [38], and all values and parameter estimates were adjusted to account for HCHS/SOL’s complex survey design using the “survey” package in R [39], with the exception of sample sizes for individual variables which are presented as unweighted.

#### 2.4.1. Population characteristics

Demographic, diet, and clinical traits measures stratified by avocado intake (consumer vs. non consumer) were calculated as a mean ( ± standard error; SE) for continuous variables, or total number (unweighted N) and percentage (%) for categorical or ordinal variables. Differences by avocado consumption were conducted using t-tests for continuous variables (transformed where necessary) and chi-squared tests of difference for categorical variables. Although unweighted counts are provided for the number of participants with each measure, weighted degrees of freedom (df) from tests of difference corrected for sampling strategy are presented throughout.

#### 2.4.2. Associations of avocado intake with glucose and insulin homeostasis

The main effect of avocado intake on each glycemic trait was examined in multivariable linear regression models, in a three-step manner. Prior to inclusion as an outcome, HbA1c and insulin and glucose values, were transformed to approximate a normal distribution, using a rank normal transformation with blom constant. All models specified avocado intake (as a continuous variable in servings/day) as the independent variable. The first set of models (“minimally adjusted” models) included age, sex, education level, total energy intake (kcal/day), acculturation score, and a term crossing field center and heritage (which are not recommended to be included as separate indicators in analyses on the HCHS/SOL population due to collinearity) as covariates. The second set of models (“fully adjusted” models) included the same covariates as the minimally adjusted models, with the addition of alcohol intake (drinks/day), physical activity, smoking status, and dietary quality (aHEI score) as covariates. If BMI is involved in any relationship between avocado intake and glycemia, whether it serves as a mediator, moderator or confounder is not clear from the existing literature. Therefore, rather than include BMI as a covariate in our main models (and potentially control for the mechanism of interest), BMI was included in an addition, final set of models (“fully adjusted + BMI” models).

The variance inflation factor (VIF) was used to measure the extent of collinearity in our regression models, with VIF values at or above 5 as the cutoff for concern for multicollinearity. All models yielded VIF values in the range of 1.077–1.702 suggesting no significant presence of multicollinearity among the covariates.

Given that our six glycemia traits were interdependent and highly correlated, significance was set at P < 0.05.

## 3. Results

### 3.1. Population characteristics

Sociodemographic, diet, and health behavior characteristics, and anthropometric and glycemic traits are presented in Table 1, stratified by avocado intake status (consumer vs. non consumer). Across the whole population, avocado consumers consumed an average of 0.36 servings ( ± 0.01) of avocado a day. Compared to non-consumers, avocado consumers had lower acculturation scores (t = −2.2, df = 639, P = 0.03, Table 1), better overall diet quality (t = 7.0, df = 639, P < 0.001, Table 1), lower BMIs (t = −4.4, df = 639, P < 0.001: Table 1), consumed more alcohol (t = 2.7, df = 239, P = 0.01, Table 1), and had a different distribution of Hispanic heritage (X^2^ = 9.7, df = 38.06, P < 0.001; Table 1). There were no differences between consumers and non-consumers in age, gender distribution, education level, physical activity, nor smoking status (all P > 0.05; Table 1).

### 3.2. Associations of avocado intake with glucose and insulin homeostasis

#### 3.2.1. Overall population

**Figure 2:**
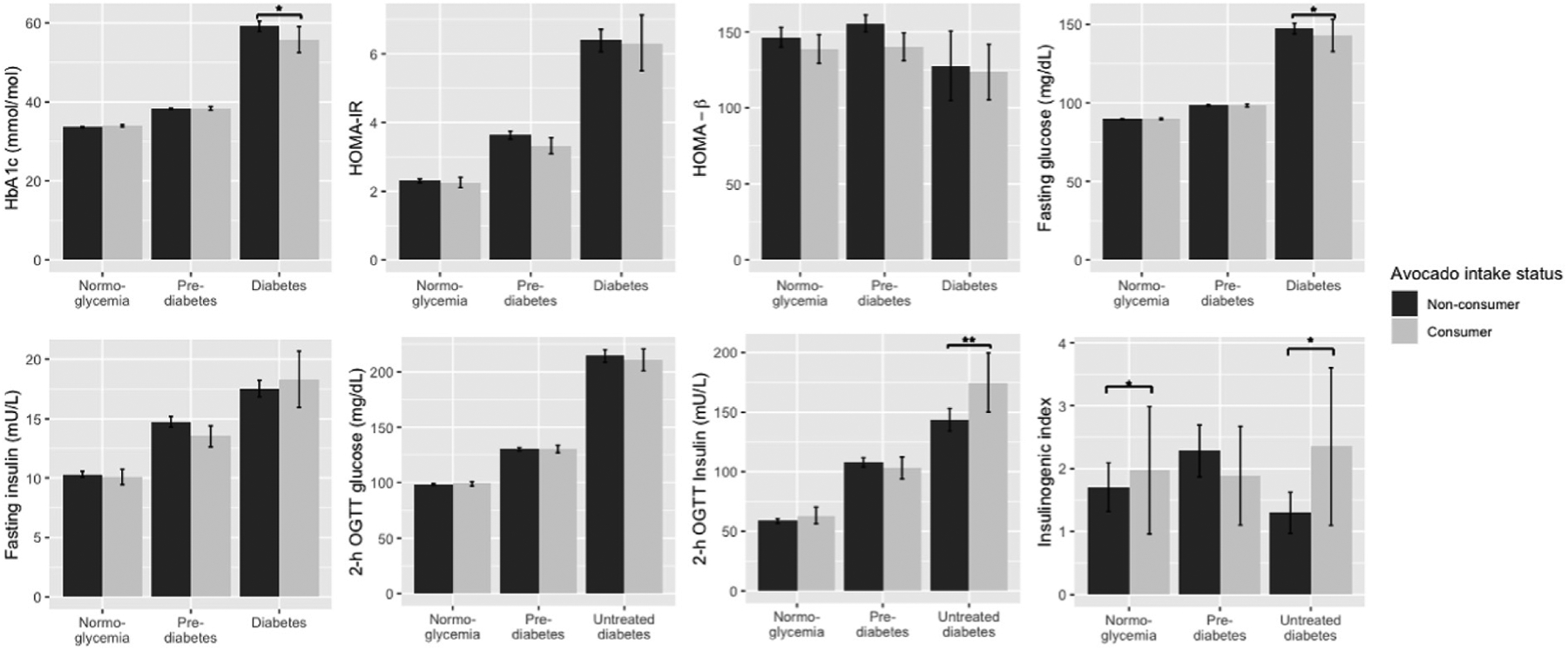
Weighted Means ( ± 95% Confidence Intervals) for Measures of Glucose and Insulin Homeostasis, Stratified by Dysglycemia Status *P < 0.05, ** P < 0.01, in models which control for age, gender, energy intake (kilocalories/day), education level, acculturation, and Hispanic/Latino heritage, alcohol intake, physical activity (metabolic equivalent minutes/day), diet quality (alternative Healthy Eating Index [aHEI] score), and smoking status (fully adjusted model), with parameter estimates weighted to reflect the complex sampling design, in models where avocado was specified as a continuous variable (servings/day).

Across the whole population, avocado intake was associated with lower HbA1c levels; each USDA serving (1/2 cup) of avocado intake was associated with HbA1c values which were 5.35 ( ± 0.47) mmol/mol lower [equivalent to 0.14% ( ± 0.04) lower %HbA1c] in the fully adjusted mode (β = −0.11, SE = 0.05, P = 0.04; Table 2; Fig. 2). Avocado intake was also associated with higher insulinogenic index scores; with each daily serving associated with scores which were 1.05 ( ± 0.54) lower in the fully adjusted model (β = −0.17, SE = 0.07, P = 0.02; Table 3; Fig. 2).

The association between avocado intake and insulinogenic index remained significant when controlling for BMI (β = −0.19, SE = 0.07, P = 0.01; Table 3), and although the inverse association with level of HbA1c was no longer significant when controlling for BMI, it remained in the same direction (β = −0.08, SE = 0.05, P = 0.15; Table 2).

#### 3.2.2. Individuals with normoglycemia

When analyses were stratified by dysglycemia, only the insulinogenic index was significantly associated with avocado intake among the participants with normoglycemia, with each serving of avocado associated with scores which were 1.81 ( ± 0.71) lower (β = 0.27, SE = 0.10, P = 0.01; Table 3; Fig. 2) in the fully adjusted model, a relationship that remained when controlling for BMI (β = 0.29, SE = 0.09, P = 0.002; Table 3).

All Individuals with T2D Among those with T2D, avocado intake was associated with lower average glucose levels; each serving of avocado intake was associated with HbA1c values which were 8.52 ( ± 2.96) mmol/mol lower [equivalent to 0.78% ( ± 0.27%) lower %HbA1c] in the fully adjusted model (β = −0.37, SE = 0.16, P = 0.02; Table 2; Fig. 2).

Fasting glucose was also inversely associated with avocado intake in participants with T2D; each daily serving of avocado was associated with fasting glucose values which were 16.01 ( ± 7.86) mg/dL lower in the fully adjusted model (β = −0.27, SE = 0.12, P = 0.02; Table 3; Fig. 2).

The inverse association between avocado intake and HbA1c in those with T2D was not altered when controlling for BMI (β = −0.37, SE = 0.16, P = 0.02; Table 2), nor was the inverse association between avocado intake and fasting glucose (β = −0.27, SE = 0.12, P = 0.02; Table 3).

#### 3.2.3. Individuals with T2D, untreated subgroup

**Figure 3:**
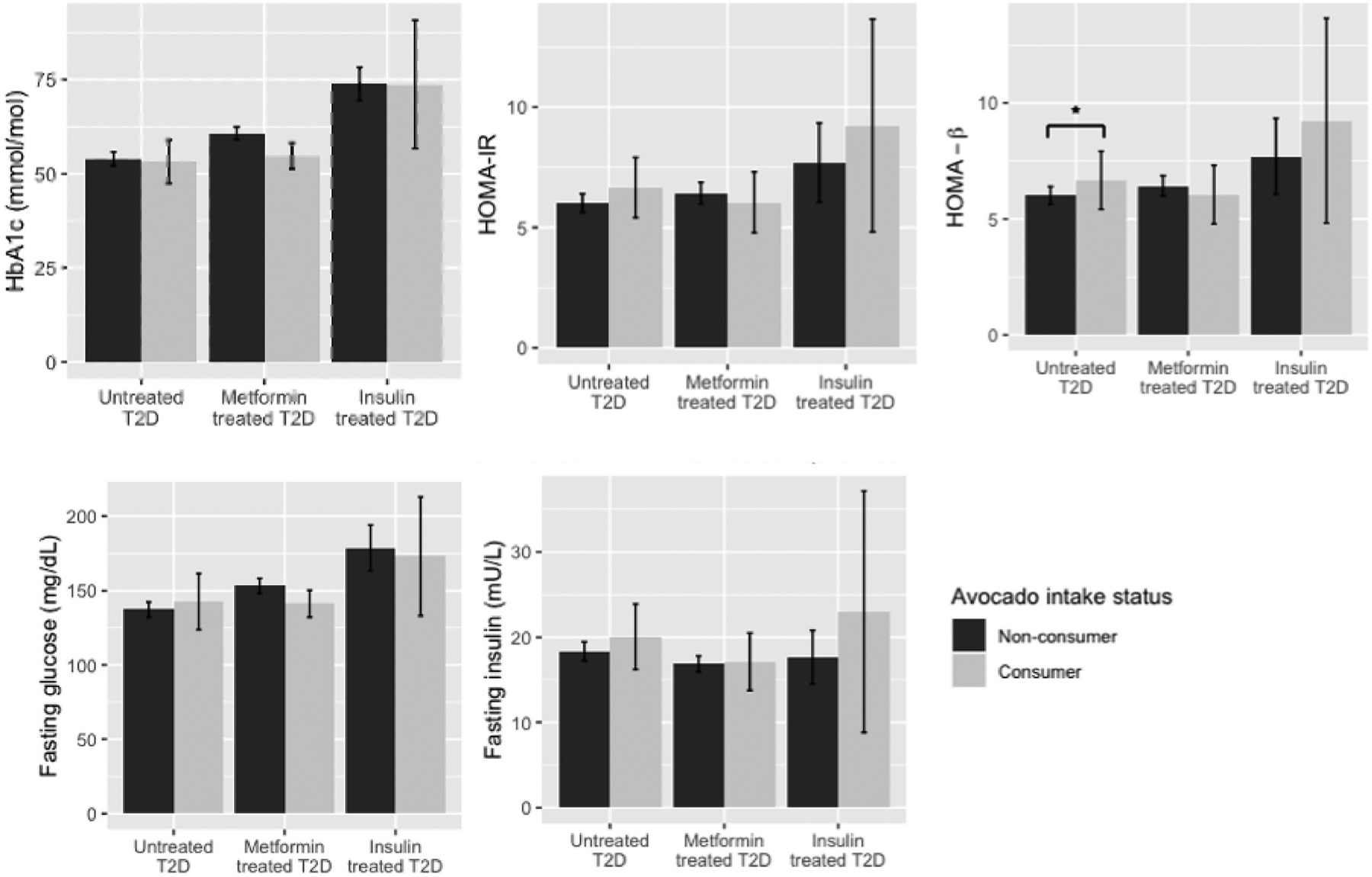
Weighted Means ( ± 95% Confidence Intervals) for Glucose and Insulin Homeostasis Measures Among Individuals with Type 2 Diabetes, Stratified by Treatment Modality *P < 0.05, in models which control for age, gender, energy intake (kilocalories/day), education level, acculturation, and Hispanic/Latino heritage, alcohol intake, physical activity (metabolic equivalent minutes/day), diet quality (alternative Healthy Eating Index [aHEI] score), and smoking status (fully adjusted model), with parameter estimates weighted to reflect the complex sampling design, in models where avocado was specified as a continuous variable (servings/day).

Although underpowered to detect significant effects, in those with untreated T2D, we observed an inverse association between avocado intake and HbA1c with a similar magnitude of effect as all participants with T2D (β = −0.36, SE = 0.21, P = 0.08; Table 2; Fig. 3).

In participants with untreated T2D, we also observed an association between avocado intake and HOMA-%β and avocado intake and insulinogenic index. Each daily serving of avocado was associated with HOMA-%β scores which were 7.95 ( ± 34.11) higher in the fully adjusted model (β = 0.39, SE = 0.04, P = 0.04; Table 3; Fig. 2). Each daily serving of avocado was associated was associated with insulinogenic index scores 2.99 ( ± 1.83) higher in the fully adjusted model (β = 0.42, SE = 0.18, P = 0.02; Table 3, Fig. 2).

The association between avocado intake and HOMA-%β was slightly attenuated when controlling BMI (β = 0.33, SE = 0.18, P = 0.06; Table 3), but the association between avocado intake and insulinogenic index remained of a similar magnitude when additionally controlling for BMI in the fully adjusted + BMI model (β = 0.43, SE = 0.17, P = 0.01; Table 3).

#### 3.2.4. Individuals with prediabetes

No associations between avocado intake and glucose or insulin homeostasis were seen in participants with prediabetes (all P > 0.05; Tables 2 and 3, Fig. 2).

## 4. Discussion

**Figure 4:**
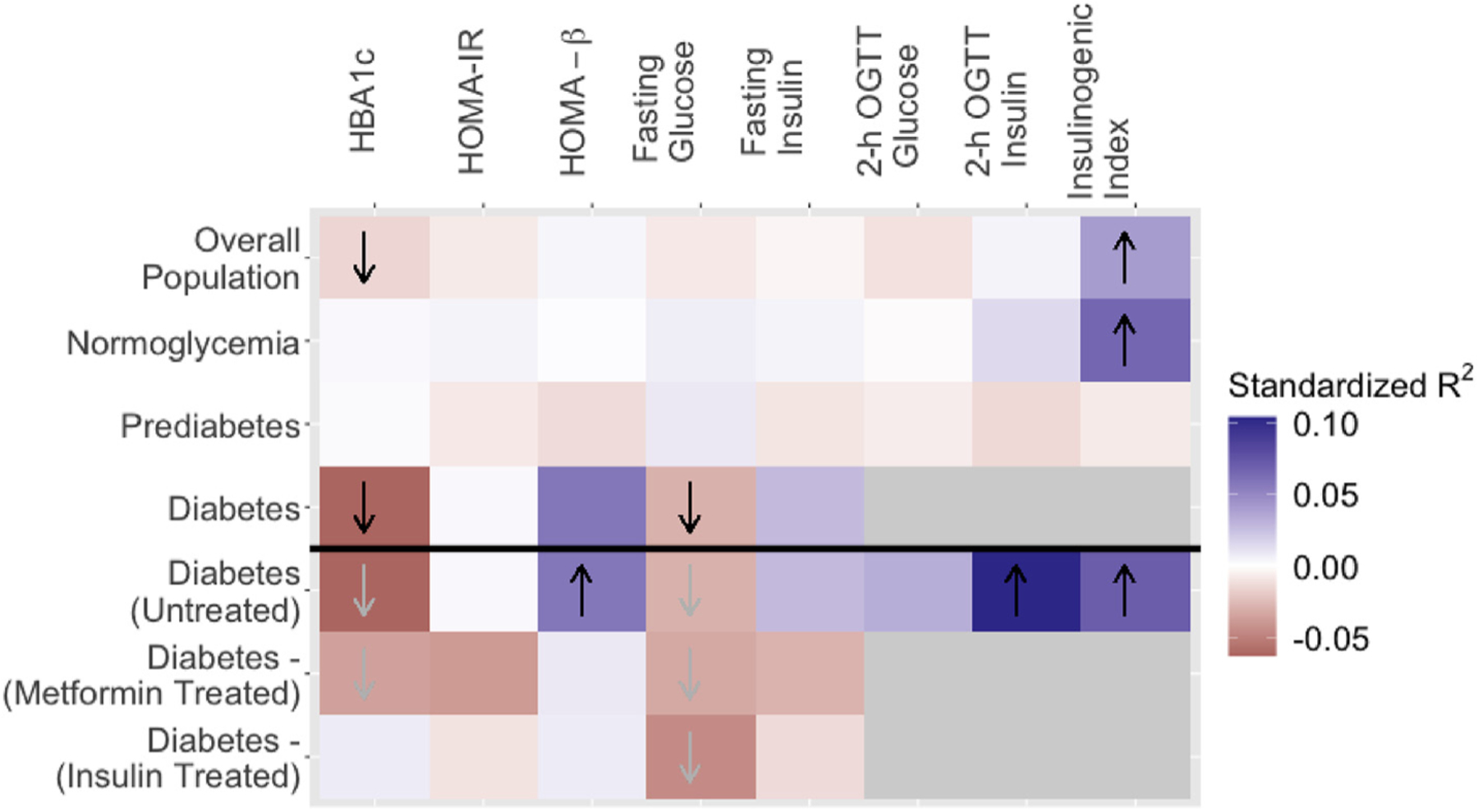
Standardized Parameter Estimates from Weighted Linear Regression Models Examining the Association of Average Avocado Intake (Servings/Day) with Glucose and Insulin Homeostasis, Stratified by Dysglycemia status *Abbreviations*: 2-h OGTT Glucose: Glucose values 2-h after the start of an oral glucose tolerance test; 2-h OGTT Insulin: Insulin values 2-h after the start of an oral glucose tolerance test HbA1c: Hemoglobin A1c; HOMA-β: Homeostatic model assessment of β-cell function; HOMA-IR: Homeostatic model assessment of insulin resistance. *Note*: All models control for age, gender, energy intake (kilocalories/day), education level, acculturation, and Hispanic/Latino heritage, alcohol intake, physical activity (metabolic equivalent minutes/day), diet quality (alternative Healthy Eating Index [aHEI] score), and smoking status. Direction of arrows equates to direction of association. Arrows in black denote significant associations (P < 0.05). For analysis by T2D subgroup (untreated vs. metformin treated vs. insulin treated) only, where there was a significant effect in the population with T2D only, arrows in grey denote similar effect sizes with the whole T2D group, for non-significant associations (P > 0.05). Grey squares indicate missing data (since participants with known T2D did not complete the OGTT).

Using data from HCHS/SOL, a large, population-based cohort of Hispanic/Latino adults, the current analyses revealed associations between avocado intake and measures of glucose and insulin homeostasis, which differed according to an individual’s dysglycemia status. The relationships we observed are summarized in Fig. 4. Among the participants with normoglycemia, avocado intake was associated with a higher insulinogenic index. In those with T2D (unless treated with insulin), avocado intake was associated with lower HbA1c values, and lower fasting glucose. In the subgroup of those with untreated T2D, avocado intake was also associated with higher HOMA-%β values, higher insulin levels 2-h after an oral glucose challenge, and a higher insulinogenic index. Despite a lack of associations in adults with prediabetes, the results here suggest that a diet containing avocado is associated with better glucose homeostasis. Our overall pattern of results also support emerging data that dietary advice should be tailored to an individual’s metabolic state (see Refs. [13,14,17]), and that optimal strategies may differ for those with vs. those without dysglycemia.

The current analyses are the first to examine associations between habitual avocado intake (i.e., for greater than 12 weeks, the length of previous intervention studies) and average glucose levels, reporting that each average daily half-cup serving of avocado is associated with HbA1c values that are ~5 mmol/mol lower in Hispanic/Latino adults. However, this inverse association was only significant in those with T2D (unless treated with insulin), in whom each serving of avocado was associated with HbA1c values that were ~7 mmol/mol lower, and not in those with normoglycemia or prediabetes. Whether this difference is enough to convey clinical benefits is not clear, but previous studies have reported that consuming avocado is associated with a lower risk of incident T2D, and in line with the current findings, this association was only found in those with prediabetes at baseline, and not in those with normoglycemia [17].

As summarized in Fig. 4, in the current study, avocado intake was associated with a higher insulinogenic index i.e., more insulin change relative to glucose change, 2-h after an oral glucose challenge in individuals with normoglycemia, and in those with T2D. In those with normoglycemia, avocado intake was not associated with higher insulin values 2-h after oral glucose, nor with higher fasting insulin, suggesting that avocado intake is only associated with higher insulin relative to plasma glucose, and so not indicative of a generally hyperinsulinemic state that could be problematic over the long term. However, in those with T2D, avocado intake was associated with a higher insulinogenic index, and higher insulin levels 2-h after an oral glucose challenge. This result converges from a small scale randomized cross-over trial, in 31 adults with overweight and/or obesity, who are more likely to have dysglycemia, had lower peak insulin and glucose concentrations after consuming a meal with avocado, when compared to consuming an energy-matched avocado free meal [15]. Although one prior RCT did not find that adding, nor substituting, avocado intake a test meal altered post-prandial insulin or glucose responses [16], this study focused on a population of adults with normoglycemia, supporting our finding that the many of the associations between avocado and glycemia were limited to those with dysglycemia (i.e., prediabetes and/or T2D). The likelihood that our observed associations between avocado intake with a higher insulinogenic index, and higher insulin levels 2-h after an oral glucose challenge, reflect compensatory increases in glucose-stimulated insulin secretion, thought to occur in response to insulin resistance [40]., is supported by a finding of higher HOMA-%β values in those with untreated T2D, suggestive of better β-cell functioning. However, a contemporary hypothesis posits that glucose toxicity is a major contributor to β-cell dysfunction in patients with T2D [41,42]. In this case, differences in β-cell function with avocado intake could arise as a consequence of lower fasting glucose levels, rather than a cause of these. Longitudinal studies, incorporating measures of insulin clearance, are warranted to disentangle the potential chain of events.

Overall, we observed that avocado intake was associated with several measures indicative of better glycemic control in those with T2D. This association could be attributable to the bioactive compounds in avocado. For example, five weeks’ supplementation with avocatin B, an avocado derived lipid, improved glucose tolerance, glucose utilization, and reduced insulin resistance in mice with diet-induced obesity [43]. This effect was attributed to reduced oxidation of fatty acids, but this mechanism was not tested, nor how this may generalize to human metabolism examined [43]. It is also not clear why bioactive compounds would be associated with glycemia in those with T2D, but not normoglycemia. Other evidence supports the overall macronutrient content of avocado as a plausible mediating mechanism, especially the high MUFA content. MUFA is thought to have a beneficial effect of dietary on insulin sensitivity via a conserved IRS-1/PI3 kinase insulin signaling pathway [44]. Although we cannot rule out the effect of bioactive compounds, such as avocatin B, attributing the beneficial associations to MUFA is attractive because, as in the current study, the association of MUFA with better glycemia is strongest in those with existing dysglycemia [13,14].

While our study benefited from the large number of participants, geographical diversity underlying their heritage, and sample weights to improve generalizability to the overarching US Hispanic/Latino population, sample weights did not account for the small amounts of missing data, nor were they available for individual strata (e.g., by sex). As missing data were considered “missing completely at random” (MCAR), the bias introduced by analyzing a subsample of participants from the overall population is likely to be negligible. However, generalizations of our conclusions to the Hispanics/Latinos population should be made with caution, especially with respect to the effect size and we encourage a focus on the overall trend in results instead. In addition, our putative risk factor, avocado, was ascertained via self-report. Our estimated avocado consumption was representative of the target population’s intake, but as avocado is an episodically consumed food even among Hispanics/Latinos, the use of two 24-h recalls may have underestimated the usual intake and misclassified some consumers as non-consumers, introducing a source of error and over above the expected errors when using self-reported dietary intake data [45]. As with all observational studies, it is not possible to ensure that all confounders (including both measured and unmeasured factors) were removed or accounted for, which precludes causal inferences; for example it is also possible that foods consumed in combination with avocados such as tomatoes and spices (as in guacamole) also have some bearing on the observed beneficial glycemic effects, or that lifestyle factors that correlate with avocado intake account for the observed associations. We also did not have the power to distinguish between individuals using insulin into subgroups according to their diabetes type, and how well controlled their dysglycemia was. Finally, due to the cross-sectional nature of our observations, each measure of glucose or insulin homeostasis was treated independently. Future studies that examine whether the effect of avocado on one glycemia parameter mediates any effect on other glycemia parameters would add valuable insights to the pattern of results found in the current analysis.

The Hispanic/Latino community in the US shows an increasing prevalence of T2D [46] and preventing the onset of chronic diseases in this population has been noted as a priority research area by the National Institutes of Health [47]. In this first study to examine associations between avocado intake and multiple measures of glycemia, including fasting and postprandial measures of insulin and glucose, as well as an integrated measure of long-term glucose homeostasis (HbA1c), we observed associations between avocado intake and higher insulin in the context of plasma glucose after an oral glucose challenge across all participants. In those with T2D only, we observed lower HbA1c values, lower fasting glucose, and higher postprandial insulin levels associated with avocado intake, and among those with untreated T2D, higher HOMA-%β. values These differences suggest that avocados may play be part of a diet aimed at supporting glucose homeostasis, but their role may be sensitive to an individual’s overall glycemia status and metabolic functioning. Future intervention/feeding studies are needed to provide more definitive evidence as to how and why avocado shows association with measures of glycemia indicative of better glucose homeostasis, and how this can be harnessed for the management, and perhaps the prevention, of T2D.

## Figures and Tables

**Table 1:** Weighted Mean ± standard deviation (SD), or Unweighted Frequency (N) and Percentage (%), for Sociodemographic Factors, Health Behaviors, and Clinical Characteristics for the HCHS/SOL population, Stratified by Avocado Intake Status (Consumers vs. Non-Consumers).

	Avocado non-consumers	Avocado consumers
*Sociodemographics*		
Age, y	41.2 (0.26)	41.5 (0.56)
Gender		
Female, N^a^ (%)	7420 (60.15%)	1412 (62.59%)
Male, N^a^ (%)	4915 (39.85%)	844 (37.41%)
*Hispanic/Latino Heritage* *		
Dominican, N^a^ (%)	1141 (9.26%)	138 (6.13%)
Central American, N^a^ (%)	1331 (10.81%)	201 (8.93%)
Cuban, N^a^ (%)	1807 (14.67%)	304 (13.50%)
Mexican, N^a^ (%)	4711 (38.25%)	1170 (51.95%)
Puerto Rican, N^a^ (%)	2115 (17.17%)	236 (10.48%)
South American, N^a^ (%)	842 (6.84%)	145 (6.44%)
More than one heritage/Other, N^a^ (%)	369 (3.00%)	58 (2.58%)
*Education level*		
No high school diploma/GED, N^a^ (%)	4653 (37.78%)	790 (35.11%)
High school diploma/GED, N^a^ (%)	3174 (25.77%)	572 (25.87%)
Greater than high school/GED, N^a^ (%)	4488 (36.44%)	878 (39.02%)
Acculturation score*	1.92 (0.04)	1.81 (0.05)
*Health Behaviours*		
Physical Activity, MET-min/day	692 (17.1)	748 (36.9)
Alcohol intake, g/day*	0.27 (0.01)	0.33 (0.02)
Diet quality, aHEI total score*	47.40 (0.17)	49.30 (0.32)
Smoking status		
Non-smoker, N^a^ (%)	10,002 (81.21%)	1871 (82.97%)
Current smoker, N^a^ (%)	2314 (18.79%)	384 (17.03)
*Clinical characteristics*		
BMI, kg/m^2^ *	29.5 (0.09)	28.6 (0.02)

*Abbreviations*: aHEI: alternative Healthy Eating Index; HCHS/SOL: Hispanic Community Health Study/Study of Latinos; MET-min: Metabolic equivalent minutes.

* P < 0.05 for differences between avocado consumers vs. non-consumers.

a Unweighted frequency.

**Table 2:** Parameter estimates from weighted regression models examining the association of average avocado intake (servings/day) with average glucose levels (defined by hemoglobin A1c).

Model	Overall Population		Normoglycemic		Prediabetic		Diabetic							
	β(SE)	P	β(SE)	P	β(SE)	P	All		Untreated		Treated			
							β(SE)	P	β(SE)	P	Metformin		Insulin	
											β(SE)	P	β(SE)	P
	N = 14,536^a^		N = 5,896^a^		N = 5,993^a^		N = 3003^a^		N = 1,495^a^		N = 1,237^a^		N = 317^a^	
1	**−0.11 (0.05)**	**.03**	−0.004 (0.05)	.94	−0.01 (0.08)	.88	**−0.37 (0.18)**	**.02**	−0.35 (0.22)	.11	−0.26 (0.17)	.12	0.07 (0.24)	.78
2	**−0.11 (0.05)**	**.04**	0.002 (0.05)	.97	−0.01 (0.08)	.94	**−0.37 (0.16)**	**.02**	−0.35 (0.21)	.10	−0.26 (0.17)	.13	0.07 (0.24)	.77
3	−0.08 (0.05)	.15	0.02 (0.06)	.83	0.01 (0.07)	.88	**−0.37 (0.16)**	**.02**	−0.36 (0.21)	.08	−0.27 (0.17)	.12	0.07 (0.23)	.76

Note: Significant results (P < 0.05) in bold.

Model 1 includes age, gender, energy intake (kilocalories/day), education level, acculturation, and Hispanic/Latino heritage as covariates (minimally adjusted model).

Model 2 includes the covariates from model 1, with the addition of alcohol intake, physical activity (metabolic equivalent minutes/day), diet quality (alternative Healthy Eating Index [aHEI] score) and smoking status (fully adjusted model).

Model 3 includes the covariates from model 2, with the addition of body mass index (BMI; fully adjusted + BMI model).

a Unweighted frequency.

**Table 3:** Parameter estimates from weighted regression models examining the association of average avocado intake (servings/day) with glucose and insulin homeostasis.

Model	Overall Population		Normoglycemic		Prediabetic		Diabetic							
	β(SE)	P	β(SE)	P	β(SE)	P	All		Untreated		Treated			
							β(SE)	P	β(SE)	P	Metformin		Insulin	
											β(SE)	P	β(SE)	P
	N = 14,555^a^		N = 5,892^a^		N = 5,419^a^		N = 3,006^a^		N = 1,498^a^		N = 1,236^a^		N = 369^a^	
HOMA-IR														
1	−0.15 (0.07)	.04	−0.07 (0.09)	.48	−0.15 (0.12)	.20	−0.17 (0.15)	.26	0.08 (0.18)	.67	−0.48 (0.21)	.02	0.05 (0.43)	.90
2	−0.11 (0.07)	.10	−0.04 (0.09)	.65	−0.10 (0.12)	.38	−0.13 (0.14)	.35	0.06 (0.17)	.71	−0.34 (0.22)	.12	0.003 (0.41)	.99
3	−0.04 (0.05)	.46	0.2 (0.07)	.77	−0.05 (0.09)	.65	−0.12 (0.12)	.23	0.02 (0.13)	.90	−0.29 (0.21)	.17	−0.10 (0.48)	.84
*HOMA-%β*														
1	−0.07 (0.07)	.30	−0.08 (0.09)	.39	−0.18 (0.13)	.16	0.14 (0.17)	.15	**0.41 (0.20)**	**.04**	−0.11 (0.24)	.66	0.06	0.57
.2	−0.04 (0.07)	.53	−0.06 (0.09)	.53	−0.12 (0.12)	.30	0.27 (0.16)	.10	**0.39 (0.19)**	**.04**	0.001 (0.24)	.99	0.14 (0.52)	.79
3	0.02 (0.05)	.71	0.004 (0.06)	.95	−0.07 (0.10)	.50	0.29 (0.15)	.05	0.33 (0.18)	.06	0.06 (0.23)	.78	0.07 (0.55)	.90
*Fasting glucose*														
1	−0.07 (0.05)	.13	0.01 (0.05)	.78	0.03 (0.08)	.69	**−0.27 (0.12)**	**.03**	−0.17 (0.15)	.23	−0.24 (0.19)	.20	−0.25 (0.60)	.68
2	−0.07 (0.05)	.15	0.02 (0.05)	.66	0.04 (0.08)	.62	**−0.27 (0.12)**	**.02**	−0.17 (0.14)	.25	−0.24 (0.18)	.20	−0.36 (0.56)	.51
3	−0.05 (0.04)	.37	0.03 (0.05)	.50	0.05 (0.08)	.55	**−0.27 (0.12)**	**.02**	−0.17 (0.14)	.23	−0.25 (0.18)	.18	−0.42 (0.54)	.44
*Fasting Insulin*														
	N = 14,517^a^		N = 5,886^a^		N = 5,411^a^		N = 3,003^a^		N = 1,493^a^		N = 1,231^a^		N = 351^a^	
1	−0.13 (0.07)	.08	−0.07 (0.10)	.47	−0.17 (0.13)	.18	−0.03 (0.16)	.82	0.23 (0.19)	.21	**−0.42 (0.20)**	**.03**	−0.01 (0.34)	.98
2	−0.10 (0.07)	.17	−0.05 (010)	.63	−0.12 (0.13)	.35	0.01 (0.15)	.98	0.22 (0.18)	.22	−0.28 (0.21)	.18	−0.03 (0.34)	.93
3	−0.02 (0.05)	.72	0.02 (0.07)	.79	−0.05 (0.10)	.61	0.03 (0.12)	.81	0.16 (0.13)	.25	−0.21 (0.19)	.27	−0.12 (0.41)	.77
*2-h OGTT Glucose*														
	N = 11,991^a^		N = 5,625^a^		N = 5,191^a^		–		N = 989^a^		–		–	
1	−0.09 (0.05)	.08	−0.03 (0.06)	.65	−0.06 (0.07)	.42	–	–	0.18 (0.17)	.31	–	–	–	–
2	−0.08 (0.05)	.11	−0.02 (0.06)	.70	−0.05 (0.08)	.54	–	–	0.19 (0.16)	.24	–	–	–	–
3	−0.05 (0.05)	.27	−0.01 (0.06	.89	−0.04 (0.08)	.64	–	–	−/20 (0.16)	.23	–	–	–	–
*2-h OGTT Insulin*														
	N = 11,966^a^		N = 5,607^a^		N = 5,183^a^		–		N = 989^a^		–		–	
1	−0.05 (0.06)	.39	0.01 (0.09)	.91	−0.15 (0.09)	.10	–	–	**0.62 (0.24)**	**.01**	–	–	–	–
2	−0.03 (0.06)	.65	0.03 (0.09)	.76	−0.11 (0.08)	.19	–	–	**0.62 (0.23)**	**.01**	–	–	–	–
3	0.02 (0.05)	.70	0.06 (0.07)	.40	−0.07 (0.08)	.34	–	–	**0.63 (0.22)**	**.004**	–	–	–	–
*Insulinogenic Index*														
1	**0.16 (0.07)**	**.03**	**0.27 (0.10)**	**.01**	−0.09 (0.10)	.37	–	–	**0.42 0.18)**	**.02**	–	–	–	–
2	**0.17 (0.07)**	**.02**	**0.27 (0.10)**	**.01**	−0.05 (0.10)	.55	–	–	**0.42 (0.18)**	**.02**	–	–	–	
3	**0.19 (0.07)**	**.01**	**0.29 (0.09)**	**.002**	−0.04 (0.09)	.66	–	–	**0.43 (0.17)**	**.01**	–	–	–	

*Note*: Significant results (P < 0.05) in bold.

*Abbreviations*: HOMA-%β: Homeostatic Model Assessment for Insulin Resistance β cell function; HOMA-IR: Homeostatic Model Assessment for Insulin Resistance; OGTT: Oral glucose tolerance test.

Model 1 includes age, gender, energy intake (kilocalories/day), education level, acculturation, and Hispanic/Latino heritage as covariates (minimally adjusted model).

Model 2 includes the covariates from model 1, with the addition of alcohol intake, physical activity (metabolic equivalent minutes/day), diet quality (alternative Healthy Eating Index [aHEI] score) and smoking status (fully adjusted model).

Model 3 includes the covariates from model 2, with the addition of body mass index (BMI; fully adjusted + BMI model).

a Unweighted frequency.
